# Effect of a water-soluble alkylene oxide copolymer based bone hemostat in cardiac surgery: a prospective, multicenter, single-arm study

**DOI:** 10.1186/s13019-026-04357-6

**Published:** 2026-06-10

**Authors:** Kentaro Yamane, Muhammad F. Masood, Shengchun Wang, Lara Micallef, Rhea Parreno, Flavia C. Morone Pinto

**Affiliations:** 1 Division of Cardiothoracic Surgery, Penn State College of Medicine, Hershey, PA USA; 2 Division of Cardiothoracic Surgery, Department of Surgery, Washington University School of Medicine in Saint Louis, Saint Louis, USA; 3 Department of Advanced Surgery, Clinical Research Strategy, Baxter Healthcare Corporation, 1 Baxter Parkway, Deerfield, IL USA; 4 Department of Clinical Development, Regulatory and Clinical Operations, Baxter Healthcare Corporation, Life Sciences Park, San Ġwann, Malta; 5 Department of Statistics, Worldwide Medical, Vantive US Healthcare LLC, Deerfield, IL USA; 6 Medical Affairs, Advanced Surgery, Baxter Healthcare Corporation, Life Sciences Park, San Ġwann, 3000 SĠN Malta

**Keywords:** Ostene, Hemostatic agents, Osteogenesis, Sternotomy, Bone hemostasis, Bone wax, Bone healing

## Abstract

**Objectives:**

This study evaluated the safety and effectiveness of an Alkylene Oxide Copolymer, Ostene, in controlling bone bleeding in open cardiac surgical patients undergoing median sternotomy.

**Design:**

Single arm, prospective, post-market study.

**Setting:**

This study was conducted at 3 centers in the United States.

**Participants:**

Ninety adult median sternotomy patients in whom Ostene was intended to be bone hemostat.

**Interventions:**

Ostene used to treat bone bleeding during surgical procedure.

**Measurements and main results:**

Surgical parameters included the area of bone bleeding, severity of bleeding, quantity of Ostene used to stop bone bleeding, time for bone bleeding to stop, time *to* occurrence of a bone rebleed, and the number of Ostene applications. The use and rationale for using an alternate bone hemostat were also recorded in cases where alternate bone hemostats were used. Adverse events that started from the time of the index procedure until 30 days postoperatively were also recorded.

**Main results:**

Intraoperative bone bleeding was successfully controlled with the application of Ostene in 87 of 89 patients (97.8%) who were eligible for effectiveness analyses. Intraoperative rebleeds were observed in 23 of 90 patients (25.6%) 238.1 ± 61.7 min after hemostasis of the original bleed was achieved. Final hemostasis was achieved by reapplying Ostene in 21 patients and alternate bone hemostats in 2 of the 23 patients. None of the patients in the study had postoperative bone bleeding within 24 h of surgery. Few AEs occurred in the study cohort, all unrelated to Ostene.

**Conclusions:**

Ostene proved to be effective bone hemostat for the control of intraoperative bone bleeding in cardiac surgical patients undergoing median sternotomy.

**Supplementary Information:**

The online version contains supplementary material available at 10.1186/s13019-026-04357-6.

## Highlights

Ostene Bone Hemostasis Material is a synthetic water-soluble alkylene oxide copolymer.

The effectiveness of Ostene was measured by evaluating the requirement for the intraoperative use of an alternate bone hemostat and by determining the number of rebleeds within 24 h.

Intraoperative bone bleeding was successfully controlled by applying Ostene.

Postoperative bone bleeding within 24 h of surgery was not reported.

Ostene proved to be effective bone hemostat for the control of intraoperative bone bleeding in cardiac surgical patients undergoing median sternotomy.

**Supplementary Information:**

The online version contains supplementary material available at 10.1186/s13019-026-04357-6.

## Introduction

Surgical bleeding, including that during sternotomy procedures, is recurrent. Thus, appropriate management is imperative to reduce the risk of postoperative complications [1–3]. In cardiothoracic surgery, patients are often at greater risk of bleeding-related complications [4]. Indeed, the re-exploration of bleeding after cardiac surgery is associated with significant morbidity and mortality [5]. 

Multiple topical hemostatic agents are readily available to surgeons, including non-absorbable bone wax, oxidized cellulose, fibrin sealants, polyethylene glycol hydrogels, cyanoacrylates, and gelatin foams [1, 6]. Despite the vast number of hemostatic agents that exist, finding a balance between bleeding and clotting remains a challenge [7]. 

Bone wax, a commonly used topical hemostatic agent for bone bleeding, controls bleeding from bone surfaces by acting as a mechanical barrier to occlude bleeding from the open channels of cancellous bone surfaces [1]. However, the use of bone wax significantly impairs bone healing and results in higher rates of inflammation [8]. Ostene Bone Hemostasis Material (Baxter Healthcare, Deerfield, Illinois) is a synthetic water-soluble alkylene oxide copolymer that works similarly to beeswax-based bone hemostatic agents [9] but does not inhibit osteogenesis or bone healing and is not associated with increased infection rates. The water-soluble nature of Ostene allows resorption from the application site within 48 h [10] and significantly reduces the incidence of inflammatory reactions [11–13]. 

This study aimed to evaluate the effectiveness and safety of Ostene in controlling bone bleeding in surgical patients undergoing median sternotomy. The rationale for conducting this study was to gather real-world evidence regarding the use of Ostene as a bone hemostatic agent in surgical procedures.

## Methods

This was a prospective, single-arm study conducted at three tertiary centers in the USA. The study population comprised of adult patients who underwent a single elective cardiac procedure that included sternotomy in which Ostene was intended to be the only bone hemostat used to treat bone bleeding during surgery. The index surgical site was defined as sternal bone and included all cut and bleeding bone(s) to which Ostene was applied. Ostene was applied to the cut sternal edges, pressing it against the bone or bleeding surface, so that, mechanically, hemostasis is achieved. Patients with ongoing infections at the site of implantation were excluded from this study.

The effectiveness of Ostene was evaluated by determining the requirement to switch to an alternate bone hemostat following one or multiple applications of Ostene, and the incidence of bone bleeding at the site(s) of Ostene application within 24 h postoperatively when no other bone hemostat was applied, being investigated through reoperation. Since this was a post-market study, surgeons were permitted to switch to alternative bone hemostatic agent if adequate homeostasis was not achieved with Ostene. Any use of, and rationale for switching to an alternate bone hemostat was recorded when Ostene was considered inadequate, including cases in which reapplication failed or when patient-specific factors (e.g., coagulation profile) warranted a change. The selection of the alternate product and decision to discontinue Ostene were made at the surgeon’s discretion.

**Fig. 1:**
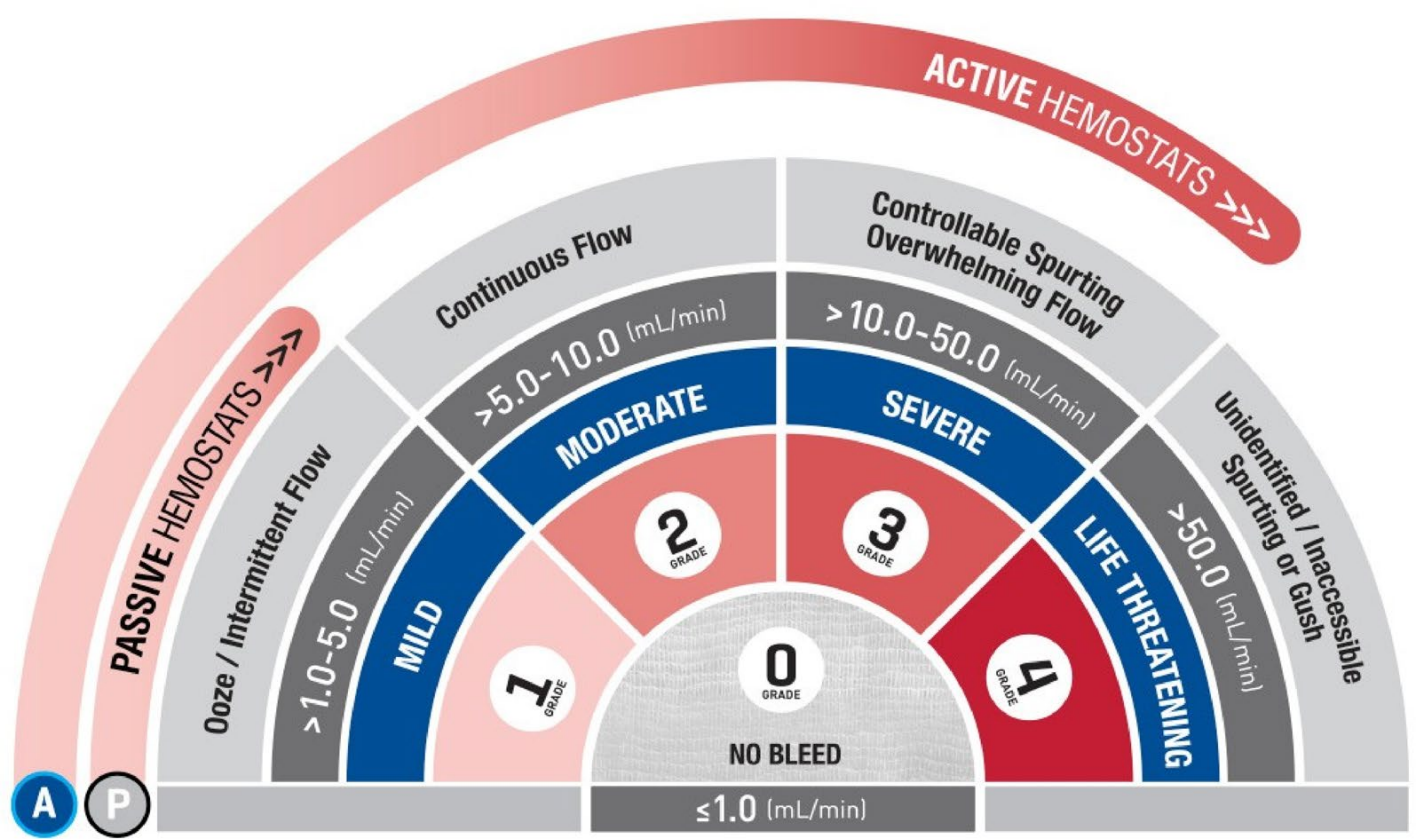
The Validated Intraoperative Bleeding (VIBe) Scale: Levels of Bleeding Severity and Types of Hemostatic Agents. Figure 1 shows the VIBe scale tool, the first surgeon-validated FDA-accepted scale, designed for consistent and reliable assessment of intraoperative bleeding severity which ranges from Grade 1 (mild) to Grade 4 (life threatening). Source: Image reproduced with permission from Baxter International

Bleeding severity was measured using the Validated Intraoperative Bleeding (VIBe) Scale (Fig. 1) [14]. The area of bone bleeding was estimated by visual examination by the surgeon [14]. This included visual cues such as speed of blood welling up after blotting with gauze, type of flow such as ooze, pulsatile, spurting, or overwhelming, and impact on the surgical field. Other surgical details that were collected and analyzed include the quantity of Ostene used, time for bone bleeding to stop, time of occurrence of bone rebleed, and number of Ostene applications.

Patients’ medical histories were collected. Two patients in this study were reported to have osteoporosis at screening. One of the two patients was the patient who had two rebleeds reported in the surgery, both of which were successfully controlled by application of Ostene. Laboratory test results related to coagulation status were collected pre-operatively and 24 h postoperatively. These included fibrinogen, partial thromboplastin time (PTT), prothrombin time (PT), prothrombin international normalized ratio (INR), and platelet count. Concomitant medication data was also collected, including blood thinners and anticoagulants taken during the 30 days prior to sternotomy and during 30 days after the procedure.

Adverse events of relevance and serious AEs (SAEs) were collected from the time of the index procedure up to 30 days postoperatively and reviewed by the surgical team. SAEs were defined according to standard criteria as events resulting in death, life-threatening conditions, hospitalization or prolongation of hospitalization, or significant medical intervention. The relatedness of SAEs to Ostene was assessed by the investigator based on clinical judgment and temporal relationship to Ostene use.

### Ethics

This study was conducted in accordance with the ethical principles based on the Declaration of Helsinki, the ethical and quality standards of good clinical practice [GCP; International Council for Harmonization (ICH) E6], International Organization for Standardization (ISO) 14155:2020, clinical investigation of medical devices for human subjects-GCP, and all applicable regulatory requirements and laws. This study was approved by the Institutional Review Board (IRB) of the participating sites. The IRB at Penn State Office for Research Protections approved IRB #65 on 25 February 2022, that at Washington University in St. Louis approved IRB #202,204,146 on 06 March 2022, and that at The Christ Hospital Health Network approved IRB #21 − 09 on 29 March 2021. Written informed consent was obtained from all patients who participated in this study.

### Statistical methods

Descriptive statistics and the exact 95% binomial confidence interval (CI) (Clopper-Pearson) for the incidence of bone bleeding at the site of Ostene application requiring the use of an alternate bone hemostat intraoperatively and for the incidence of bone bleeding being the cause of re-operation within 24 h postoperatively (when no other bone hemostat was applied) were calculated. Exploratory endpoints in this study included time for bleeding to stop, time of occurrence of a bone rebleed, area of bone bleeding, severity of bone bleeding, and quantity of Ostene used.

The exploratory endpoints were analyzed descriptively by bleeding event and using *n*, mean, standard deviation (SD), median, minimum and maximum values. The categorical endpoint was analyzed descriptively by bleeding event and using frequencies and percentages. The bleed details and reasons for use of an alternate hemostat to achieve hemostasis intraoperatively following the use of Ostene were described.

Only AEs of relevance and SAEs that started on or after the use of Ostene were summarized, including the number and percentage of patients, the number of events, and the number of events per patient.

## Results

**Fig. 2:**
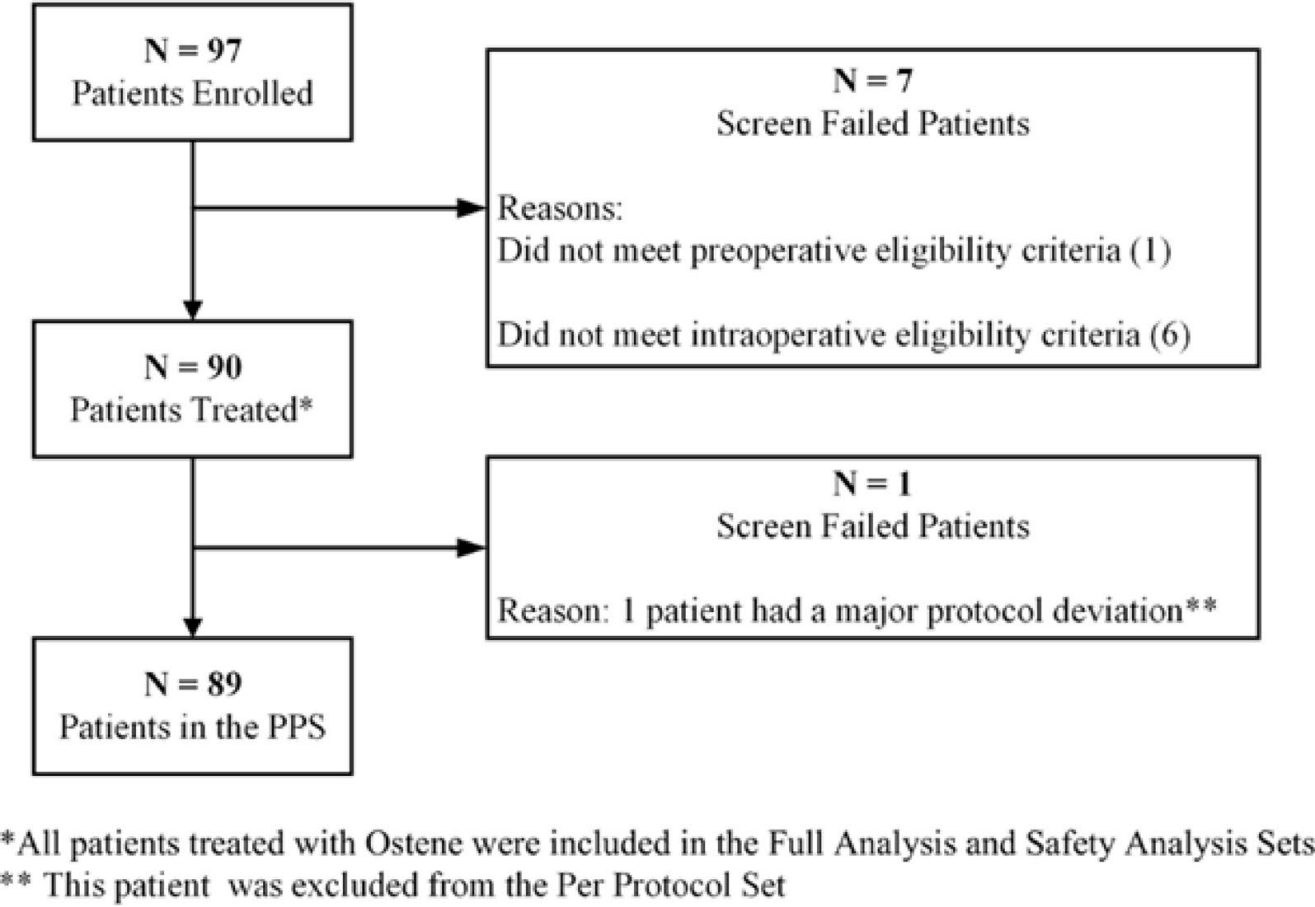
Patient Disposition Tree. Shows the screening of patients according to the following protocol steps: Patients enrolled, treated, and included in the per protocol set

Ninety-seven patients were enrolled across the three centers. Six patients screen-failed because Ostene was not used intraoperatively, and one patient consented but did not have surgery. Ninety patients were treated with Ostene, of whom 89 patients were included in the analysis of the effectiveness endpoint. One patient was excluded due to a major protocol deviation (Fig. 2).

Approximately two-thirds of the patient population was male, with a mean age of 64.2 years and a mean body mass index (BMI) of 30.6, placing the average participant within the overweight to obese category, consistent with that of typical cardiac surgery populations [15, 16] (Table 1).

**Table 1:** Patient Demographics and Baseline Characteristics

Characteristics	Category	Total (*N* = 90) *n* (%)
Sex	Female	29 (32.2)
	Male	61 (67.8)
Age (years)	Mean (SD)	64.2 (11.1)
	Median (Min; Max)	64.5 (25, 85)
Body Mass Index (kg/m^2^)	Mean (SD)	30.6 (7.0)
	Median (Min; Max)	29.6 (17.9, 52.5)
Tobacco Use	Current	12 (13.3)
	Previous	28 (31.1)
	Never	50 (55.6)
Medical History in ≥ 15% of Patients by Preferred Term	Hypertension	70 (77.78)
	Hyperlipidemia	58 (64.44)
	Coronary artery disease	55 (61.11)
	Diabetes	38 (42.22)
	Gastroesophageal reflux disease	24 (26.67)
	Aortic valve disease	19 (21.11)
	Sleep apnea syndrome	16 (17.78)
	Mitral valve disease	15 (16.67)

Forty-eight (53.3%) patients were admitted with coronary artery disease (CAD) as the primary preoperative diagnosis (Table 2). Valvular heart diseases were the second most prevalent preoperative diagnoses, followed by abnormalities of the ascending aorta or a combination of coronary and structural heart diseases.

**Table 2:** Preoperative Diagnosis/Reason for Surgery

Preoperative Diagnosis	Total (*N* = 90) *n* (%)
CAD	48 (53.3)
Aortic Valve	12 (13.3)
Mitral/Tricuspid	11 (12.2)
Aortic Valve + Ascending Aorta	6 (6.7)
Ascending Aorta	6 (6.7)
CAD + Aortic Valve	4 (4.4)
CAD + Mitral/Tricuspid	2 (2.2)
Aortic Valve + Mitral/Tricuspid	1 (1.1)

CAD = Coronary artery disease

The effectiveness of Ostene was measured by evaluating the requirement for the intraoperative use of an alternate bone hemostat and by determining the number of rebleeds within 24 h postoperatively where only Ostene was applied. Eighty-nine (98.9%) of 90 patients were included in the effectiveness analyses. One patient was excluded from the analysis as an alternate bone hemostat was used without considering the performance and effectiveness of Ostene.

In all 90 patients treated with Ostene, hemostasis of the original bone bleed was achieved in 68.8 ± 39.3 s (Table 4). Two (2.25%) of the 89 patients required the application of an alternate bone hemostat (Clopper-Pearson Exact 95% CI: 0.27%, 7.88%) as shown in Table 3. In one patient, the original bleed was severe, had an area of 8 cm^2^, and was initially controlled using 5 g of Ostene. Severe rebleeding occurred 288.2 min after hemostasis, for which an additional 5 g of Ostene was used but failed to control rebleeding. The surgeon used 5 g of bone wax to achieve hemostasis of the rebleed. The switch was made as the patient was osteoporotic and coagulopathic. The other patient had an initial moderate bleed having an area of 4 cm^2^ for which hemostasis was achieved by the application of 7.5 g of Ostene. Rebleeding occurred 285 min after initial hemostasis was achieved and was treated using an additional 5 g of Ostene, followed by 2.5 g of bone wax to achieve hemostasis. The severe and moderate bleeding events which occurred in the two patients described previously occurred intraoperatively. None of the 87 patients who received Ostene as the only bone hemostat experienced bleeding at the target site within 24 h postoperatively (Clopper-Pearson Exact 95% CI: 0.00%, 4.15%) as shown in Table 3.

**Table 3:** Incidence of Switching to Alternate Bone Hemostat and Postoperative Rebleeds

Parameter	Category	Total (*N* = 89) *n* (%)	Clopper-Pearson Exact 95% CI
Intraoperative use of an alternate bone hemostat^1^	Yes	2 (2.25)	(0.27%, 7.88%)
	No	87 (97.75)	
Postoperative bone bleeding within 24 h of surgery	Yes	0	(0.00%, 4.15%)
	No	87 (100.00)	

CI = Confidence interval

^1^An alternate bone hemostat was used due to the intraoperative underperformance/ineffectiveness of Ostene

For the 90 patients treated with Ostene, the original bone bleed had an area of 8.7 ± 6.9 cm^2^, required the application of 4.6 ± 1.6 g of Ostene, and took 68.8 ± 39.3 s to achieve initial hemostasis. Bleeding severity was measured using the VIBe scale, which ranges from 0 (no bleeding) to 4 (life-threatening). Most of the original bleeds were categorized as mild (47.8%) or moderate (43.3%) (Fig. 1) as indicated in Table 4.

Rebleeds occurred in 23 out of 90 patients (25.6%) and occurred 238.1 ± 61.7 min after the initial bleed had achieved hemostasis. The first rebleed had an average area of 4.0 ± 1.4 cm^2^, with a severity of mostly mild (52.2%) or moderate (43.5%). In 21 of the 23 patients (91.3%), rebleeding was successfully controlled by the application of additional Ostene. One patient experienced a second rebleed during surgery, 33.6 min after the first rebleed, which was controlled by an additional application of 5 g of Ostene. In total, surgeons opted to switch to an alternate hemostat in 3 patients. For 2 patients, the surgeon switched hemostat after 2 attempts with Ostene, as described above. The third patient was excluded from the effectiveness analysis because the switch was done due to surgeon preference and was unrelated to the effectiveness of Ostene.

**Table 4:** Characteristics of Intraoperative Bleeding/Rebleeding and Application of Ostene

Parameter	Category	Original Bleed (*N* = 90)	Rebleed 1 during Surgery (*N* = 23)	Rebleed 2 during Surgery (*N* = 1)
Time for bleeding to stop (s)	n Mean (SD) Median (Min; Max)	90 68.8 (39.30) 57.0 (11; 290)	22 59.0 (26.96) 50.0 (28; 122)	1 51.0 (-) 51.0 (-)
Time of occurrence of a bone rebleed (mins)	n Mean (SD) Median (Min; Max)	NA	23 238.1 (61.65) 226.7 (108.4; 355.7)	1 33.6 (-) 33.6 (-)
Area of bone bleeding (cm^2^)	n Mean (SD) Median (Min; Max)	90 8.7 (6.86) 6.0 (1; 39.6)	23 4.0 (1.38) 4.0 (2; 8)	1 3.0 (-) 3.0 (-)
Severity of bone bleeding [n (%)]^1^	0-No bleed 1-Mild 2-Moderate 3-Severe 4-Life Threatening	1 (1.1) 43 (47.8) 39 (43.3) 7 (7.8) 0	0 12 (52.2) 10 (43.5) 1 (4.3) 0	0 1 (100) 0 0 0
Quantity of Ostene used (g)	n Mean (SD) Median (Min; Max)	90 4.6 (1.59) 5.0 (2.5; 10)	23 3.4 (1.22) 2.5 (2.5; 5)	1 5.0 (-) 5.0 (-)

SD = Standard deviation; Min = Minimum; Max = Maximum

^1^The severity of bone bleeding was assessed using the VIBe Scale (Fig. 1)

In total, 18 SAEs were reported in 13 (14.4%) patients as shown in Table E1. One patient was reported to have mediastinal abscess which was assessed by the investigator as probably related to the study procedure. The clinician further assessed the event as not related to the use of Ostene but did not rule out the potential relatedness to other implantable materials and soft tissue hemostats used throughout the procedure. A total of 4 infections and infestations were recorded. These included a case of localized infection, a mediastinal abscess, a post-operative wound infection, and sepsis. None of these events were deemed to be related to Ostene by the investigator. There was 1 death in the study, assessed as unrelated to Ostene. The patient died 3 days postoperatively due to severe multiple organ failure due to pre-existing severe visceral and peripheral vascular occlusion and stenosis.

## Discussion

The management of bone bleeding is an important factor in determining a successful outcome during surgeries involving bone. Surgeons have multiple options to control bone bleeding during surgery. These include water insoluble products such as bone wax, gelatin foam, microfibrillar collagen, and oxidized cellulose [1], as well as water-soluble products like Ostene and BoneSeal Bone Hemostat (Hemostasis, LLC, Saint Paul, Minnesota) [17]. All methods used to control bone bleeding are associated with clinical benefits and risks. Thus, the surgeon must select the best option based on the individual patient’s needs.

Conventional bone wax is one of the most commonly used agents to achieve hemostasis in various surgical procedures. Bone wax is comprised primarily of beeswax and other insoluble components which remain at the site of application for a prolonged period [18]. The continued presence and physical compression of bone wax at the application site have been associated with complications including allergic reactions, foreign body granuloma [19], infection and inflammation [20], inhibition of osteogenesis, cerebrospinal fluid leakage in cranial surgeries [21], and neurological defects in spinal surgeries [20]. 

Unlike bone wax, Ostene is composed of a hydrophilic, water-soluble alkylene oxide copolymer which has a functional lifetime ranging between 2 and 5 h after application depending on the rate of wound irrigation, and is fully resorbed within 30 days [22]. Pre-clinical and clinical studies in different types of bone defects have demonstrated that Ostene results in lower rates of postoperative bone infection and inflammation compared to bone wax [23], and does not inhibit bone healing [12, 24]. The potential clinical benefit that Ostene offers in the control of bone bleeding is intrinsic to its chemical and biological properties and is therefore generally applicable irrespective of anatomical location and bone surfaces exposed under different surgical procedures [13]. 

Due to its clinical characteristics and potential in avoiding the AEs implicated with bone wax, Ostene has emerged as substitute for bone wax to control bone bleeding from surgically or traumatically fractured bone [25, 26]. Recent studies demonstrated that Ostene does not inhibit bone healing in patients who undergo sternotomy in cardiothoracic surgeries [11], and has been shown to facilitate the reduction of peridural fibrosis complications (adhesion) in patients receiving spinal laminectomy [27]. Pathological wound healing, termed fibrosis, consists of the activation of local tissue fibroblasts which, in turn, increases their contractility, the secretion of inflammatory mediators, and the synthesis of extracellular matrix components, leading to peridural fibrosis (adhesion) [28]. Ostene appears to mitigate the inflammatory response and reduces the formation of fibrosis [27]. 

Due to the close proximity to the heart, aorta, cardiac prostheses, and associated anastomosis, successful management of bone bleeding during sternotomy is crucial to improve clinical outcomes in cardiovascular procedures [9]. Bleeding control in sternotomy procedures often requires the use of a large quantity of the bone hemostat on the sternal side to keep the operative field dry, avoid excessive blood transfusions during surgery, and prevent reoperation [29, 30]. It is also imperative for the bone hemostat not to interfere with sternal bone healing as sternal dehiscence after median sternotomy can lead to catastrophic complications [31]. 

This study was the first to thoroughly evaluate the safety and effectiveness of Ostene as a bone hemostat in a multicenter cohort of 90 cardiac patients with median sternotomy. The study cohort had a 2:1 ratio of males to females with an average BMI of 30.6 ± 7.0 kg/m^2^. Patients were primarily diagnosed with CAD or structural valvular disorders and were overall representative of the cardiac population in the USA [32]. The application of Ostene resulted in hemostasis of the cut sternum in approximately 1 min for both the original bleed, (68.8 ± 39.3 s), and the rebleed (59.0 ± 27.0 s).

Serious AEs were observed in 14.4% of the study population and the most reported were cardiac disorders (5.6%), infection (4.4%), and respiratory disorders (5.6%) which were expected in a cardiac surgery patient population. Importantly, no SAEs were considered related to Ostene by the surgical team confirming its favorable. safety profile as a bone hemostat through the early postoperative phase. There were two cases in which surgeons elected to switch to an alternate bone hemostat, likely attributed to the underlying anticoagulated state characteristic of the cardiac surgery patient cohort. Note that successful hemostasis following the use of an alternative agent should be interpreted as evidence of rescue efficacy rather than superiority, as the study was not designed to compare hemostatic agents under equivalent conditions. A relatively low percentage (25%) (23 of 90) of the patients experienced a rebleed approximately 4 h after hemostasis of the original bleed. Despite this, most rebleeds were successfully controlled by the reapplication of Ostene to the bone surfaces as needed to achieve final hemostasis prior to closure. The water-soluble characteristic of Ostene may have increased the possibility of it being washed off the edge of the sternum by continuous irrigation or bodily fluids off the surgical site. This might be expected and exacerbated by the lengthy procedure time incurred in this surgical cohort. This is an expected trade-off of the hydrophilic property of Ostene which may sometimes necessitate multiple applications in long surgeries and/or extensive irrigation. Ostene acts as a mechanical (passive) hemostat, forming a physical barrier over bleeding bone surfaces. It facilitates platelet activation and aggregation, providing a matrix that supports clot formation while relying on the patients’ intrinsic coagulation status for hemostasis. As a non-biologically active agent, Ostene is best suited for patients with a responsive coagulation cascade and support secondary hemostasis rather than overriding coagulopathy [33]. In the acute postoperative phase, none of the patients in this study experienced postoperative bleeding at the site(s) of Ostene application within 24 h of surgery, indicating continued effectiveness after closure. It is important to note that reapplication of Ostene or the use of alternative hemostatic agents/methods may be appropriate in cases of severe bleeding.

## Limitations

The study protocol was originally intended to evaluate the performance and safety of Ostene across cardiac, cranial and spinal surgical procedures. A limitation of the study is that the enrolled population consisted exclusively of cardiothoracic surgery patients undergoing median sternotomy. Cardiothoracic surgeries are inherently associated with a higher risk of bleeding, particularly due to anticoagulation and procedure duration, and therefore represent a stringent, worst-case scenario for evaluating a bone hemostatic agent such as Ostene. While the findings support the safety and effectiveness of Ostene in this high-risk context, it is recognized that the result may not exhaustively represent the unique circumstances encountered during other types of surgery. The study was designed as a single-arm study that did not include a control bone hemostat group for comparative analysis. The current study was not aimed to specifically assess the effect of bone hemostats on radiologically confirmed healing. Since no objective threshold was set for the application of an alternate bone hemostat, this was done at the discretion of the surgeon. Data on delayed sternal closure was not collected, and post-operative bleeding was assumed to be absent as long as no re-intervention was warranted. The study also had a relatively short postoperative follow-up period that was not sufficient to evaluate the long-term safety and effectiveness of Ostene. Moreover, overall surgical risk and patient stratification were not assessed. Ostene was not compared with other hemostatic agents in this study; therefore, a comparative assessment of rebleeding rates is not possible. Rebleeding events were evaluated descriptively, and no causal attribution to Ostene can be scientifically verified. Future studies should aim to address these limitations.

## Conclusion

The findings of this study reflect the overall expected effectiveness of Ostene. The clinical evidence collected in a prospective, multi-center manner in a real-world setting supports the use of Ostene as an effective bone hemostat for the control of intraoperative bone bleeding in cardiac surgeries. Due to the short duration of the study, definitive long-term safety conclusions for Ostene cannot be drawn from these data alone.

## Supplementary Information

Supplementary Material 1

## Data Availability

No datasets were generated or analysed during the current study.

## Abbreviations

AE: Adverse event
BMI: Body Mass Index
CAD: Coronary artery disease
CI: Confidence interval
GCP: Good Clinical Practice
ICH: International Council for Harmonization
IRB: Institutional Review Board
ISO: International Organization for Standardization
IV: Intravenous
SAE: Serious adverse event
SD: Standard deviation
USA: United States of America
VIBe: Validated Intraoperative Bleeding
