# Supplementary material for: Effect of a water-soluble alkylene oxide copolymer based bone hemostat in cardiac surgery: a prospective, multicenter, single-arm study
Source: J Cardiothorac Surg. 2026 Jun 10;21:588. doi: 10.1186/s13019-026-04357-6 (PMC13483741; doi:10.1186/s13019-026-04357-6)
Supplement: Supplementary file 1 [file 13019_2026_4357_MOESM1_ESM.docx]

**SUPPLEMENTARY MATERIAL**

Table E1. Summary of Serious Treatment Emergent Adverse Events

| MedDRA Terminology^1^ | | Total  (N = 90) | | |
| --- | --- | --- | --- | --- |
| MedDRA  System Organ Class | MedDRA  Preferred Term | Number of Patients  n (%) | Number of Events | Number of Events per Patient |
| OVERALL | OVERALL | 13 (14.44) | 18 | 0.20 |
| Cardiac disorders | OVERALL | 5 (5.56) | 6 | 0.07 |
|  | Arrhythmia | 1 (1.11) | 1 | 0.01 |
|  | Atrial fibrillation | 2 (2.22) | 2 | 0.02 |
|  | Cardiac tamponade | 1 (1.11) | 1 | 0.01 |
|  | Pericardial effusion | 1 (1.11) | 1 | 0.01 |
|  | Supraventricular   tachycardia | 1 (1.11) | 1 | 0.01 |
| Gastrointestinal disorders | OVERALL | 1 (1.11) | 1 | 0.01 |
|  | Pneumoperitoneum | 1 (1.11) | 1 | 0.01 |
| General disorders | OVERALL | 1 (1.11) | 1 | 0.01 |
|  | Multiple organ   dysfunction   syndrome | 1 (1.11) | 1 | 0.01 |
| Infections and infestations | OVERALL | 4 (4.44) | 4 | 0.04 |
|  | Localized infection | 1 (1.11) | 1 | 0.01 |
|  | Mediastinal abscess | 1 (1.11) | 1 | 0.01 |
|  | Post-operative   wound infection | 1 (1.11) | 1 | 0.01 |
|  | Sepsis | 1 (1.11) | 1 | 0.01 |
| Injury, poisoning, and procedural complications | OVERALL | 1 (1.11) | 1 | 0.01 |
|  | Seroma | 1 (1.11) | 1 | 0.01 |
| Respiratory, thoracic, and mediastinal disorders | OVERALL | 5 (5.56) | 5 | 0.06 |
|  | Hemothorax | 2 (2.22) | 2 | 0.02 |
|  | Pleural effusion | 2 (2.22) | 2 | 0.02 |
|  | Respiratory failure | 1 (1.11) | 1 | 0.01 |
| 1 MedDRA (Medical Dictionary for Regulatory Activities) is a clinically validated international medical terminology used by regulatory authorities and the regulated biopharmaceutical industry. | | | | |
